# Supplementary material for: A Critical View of the Application of the APEX Software (Aqueous Photochemistry of Environmentally-Occurring Xenobiotics) to Predict Photoreaction Kinetics in Surface Freshwaters
Source: Molecules. 2019 Dec 18;25(1):9. doi: 10.3390/molecules25010009 (PMC7017383; doi:10.3390/molecules25010009)
Supplement: Supplementary file 1 [file molecules-25-00009-s001.zip › 3.2.4_gcc-4.4.0.zip!/3.2.4_gcc-4.4.0/3.2.4_gcc-4.4.0/doc/octave/html/interpreter/Binary-I_002fO.html]

Binary I/O - Untitled


Next: Temporary Files,
Previous: String Input Conversions,
Up: C-Style I/O Functions


---

#### 14.2.16 Binary I/O

Octave can read and write binary data using the functions `fread`
and `fwrite`, which are patterned after the standard C functions
with the same names. They are able to automatically swap the byte order
of integer data and convert among the supported floating point formats
as the data are read.

— Built-in Function: [val, count] = **fread** (fid, size, precision, skip, arch)  
> Read binary data of type precision from the specified file ID
> fid.
>
> The optional argument size specifies the amount of data to read
> and may be one of
>
> `Inf`: Read as much as possible, returning a column vector. nr: Read up to nr elements, returning a column vector. `[`nr`, Inf]`: Read as much as possible, returning a matrix with nr rows. If the number of elements read is not an exact multiple of nr, the last column is padded with zeros. `[`nr`,` nc`]`: Read up to nr `*` nc elements, returning a matrix with nr rows. If the number of elements read is not an exact multiple of nr, the last column is padded with zeros.
>
> If size is omitted, a value of `Inf` is assumed.
>
> The optional argument precision is a string specifying the type of
> data to read and may be one of
>
> `"schar"` `"signed char"`: Signed character. `"uchar"` `"unsigned char"`: Unsigned character. `"int8"` `"integer*1"`: 8-bit signed integer. `"int16"` `"integer*2"`: 16-bit signed integer. `"int32"` `"integer*4"`: 32-bit signed integer. `"int64"` `"integer*8"`: 64-bit signed integer. `"uint8"`: 8-bit unsigned integer. `"uint16"`: 16-bit unsigned integer. `"uint32"`: 32-bit unsigned integer. `"uint64"`: 64-bit unsigned integer. `"single"` `"float32"` `"real*4"`: 32-bit floating point number. `"double"` `"float64"` `"real*8"`: 64-bit floating point number. `"char"` `"char*1"`: Single character. `"short"`: Short integer (size is platform dependent). `"int"`: Integer (size is platform dependent). `"long"`: Long integer (size is platform dependent). `"ushort"` `"unsigned short"`: Unsigned short integer (size is platform dependent). `"uint"` `"unsigned int"`: Unsigned integer (size is platform dependent). `"ulong"` `"unsigned long"`: Unsigned long integer (size is platform dependent). `"float"`: Single precision floating point number (size is platform dependent).
>
> The default precision is `"uchar"`.
>
> The precision argument may also specify an optional repeat
> count. For example, 32\*single causes `fread` to read
> a block of 32 single precision floating point numbers. Reading in
> blocks is useful in combination with the skip argument.
>
> The precision argument may also specify a type conversion.
> For example, int16=>int32 causes `fread` to read 16-bit
> integer values and return an array of 32-bit integer values. By
> default, `fread` returns a double precision array. The special
> form \*TYPE is shorthand for TYPE=>TYPE.
>
> The conversion and repeat counts may be combined. For example, the
> specification 32\*single=>single causes `fread` to read
> blocks of single precision floating point values and return an array
> of single precision values instead of the default array of double
> precision values.
>
> The optional argument skip specifies the number of bytes to skip
> after each element (or block of elements) is read. If it is not
> specified, a value of 0 is assumed. If the final block read is not
> complete, the final skip is omitted. For example,
>
> ```
>           fread (f, 10, "3*single=>single", 8)
> ```
>
> will omit the final 8-byte skip because the last read will not be
> a complete block of 3 values.
>
> The optional argument arch is a string specifying the data format
> for the file. Valid values are
>
> `"native"`: The format of the current machine. `"ieee-be"`: IEEE big endian. `"ieee-le"`: IEEE little endian. `"vaxd"`: VAX D floating format. `"vaxg"`: VAX G floating format. `"cray"`: Cray floating format.
>
> Conversions are currently only supported for `"ieee-be"` and
> `"ieee-le"` formats.
>
> The data read from the file is returned in val, and the number of
> values read is returned in `count`
>
>
>
>
> **See also:** fwrite, fopen, fclose.

— Built-in Function: count = **fwrite** (fid, data, precision, skip, arch)  
> Write data in binary form of type precision to the specified file
> ID fid, returning the number of values successfully written to the
> file.
>
> The argument data is a matrix of values that are to be written to
> the file. The values are extracted in column-major order.
>
> The remaining arguments precision, skip, and arch are
> optional, and are interpreted as described for `fread`.
>
> The behavior of `fwrite` is undefined if the values in data
> are too large to fit in the specified precision.
>
>
>
> **See also:** fread, fopen, fclose.
